# Paraperesis: a rare complication after depressed skull fracture

**Published:** 2012-08-14

**Authors:** Ali Asmat Syed, Anjum Arshad, Khatoon Abida, Sardha Minakshi

**Affiliations:** 1 Jawaharlal Nehru Medical College, Aligarh Muslim University, Uttar Pradesh, India

**Keywords:** Fracture, paraperesis, edema, depressed fracture, skull

## Abstract

Depressed skull fracture is an inward buckling of the skull bones, often because of direct blow to a small surface area of the skull with a blunt object. Monoparesis is often among its clinical presentations, but midline depressed skull fracture presenting as motor weakness of both lower limbs (Paraperesis) has not yet been reported. We report the case of 55 year old male admitted to emergency department with alleged history of hit on head by a wooden rod. He had pain, mild swelling and a small cut over scalp without any symptoms & signs of neurological deficit. On day two of admission patient developed weakness of both lower limbs. On CT scan patient had bilateral depressed skull fracture of high parietal bone on either side of midline. Patient was managed conservatively, made remarkable recovery and was discharged after 2 weeks.

## Introduction

Head injury is a leading cause of mortality and morbidity particularly in developing countries. It usually manifests as skull fracture, which is a break in the cranial bones. It is common after fall from height, road traffic accidents, physical assault and other injuries [1]. The incidence of skull fractures among head injured adults who present to emergency departments (ED) is unknown. The parietal bone is most frequently fractured, followed by the temporal, occipital, and frontal bones [2]. Skull fractures are grossly classified into linear, depressed and comminuted types. Linear fractures are the most common, followed by depressed skull fractures [3]. A skull fracture is considered depressed, when any portion of the outer table of the of fracture and depth of fracture line lies below the normal anatomical position of the inner table. Depressed skull fractures typically occur when objects with a large amount of kinetic energy make contact with the skull over a fairly small area [4]. Most of the depressed fractures are over the front-parietal region (75%) as the bone is thin and the site is more prone to physical assault [5].

## Patient and observation

A 55-year old man, was hit on head by a wooden rod, he was brought within 1 hour of the alleged physical assault. He complained of pain, swelling and cut over scalp without any history of nausea, vomiting, loss of consciousness and other symptoms of head injury. On examination, his Glasgow Coma Scale (GCS) was 15/15, pupils were bilateral equal and reacting. He had no signs of neurological deficit. On local examination he had redness, swelling and laceration 1 x 1 cm over left high parietal region. Patient was managed conservatively and kept for 24 hours observation, in line with the protocol for head injury cases. Next day, patient complained of diffuse headache followed by difficulty in walking.

**Figure 1:**
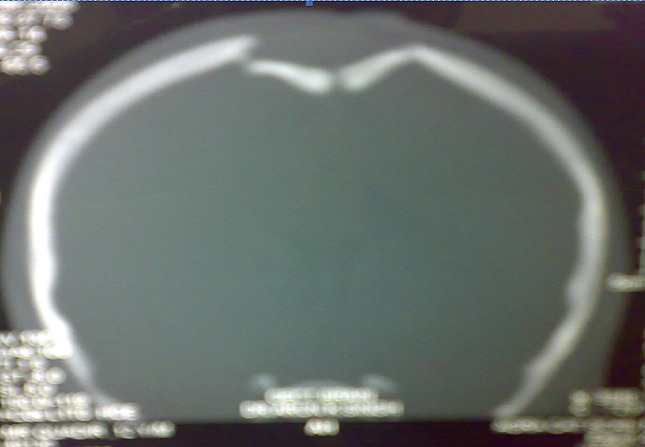
CT scan of the head showing bilateral depressed parietal bone fracture

**Figure 2:**
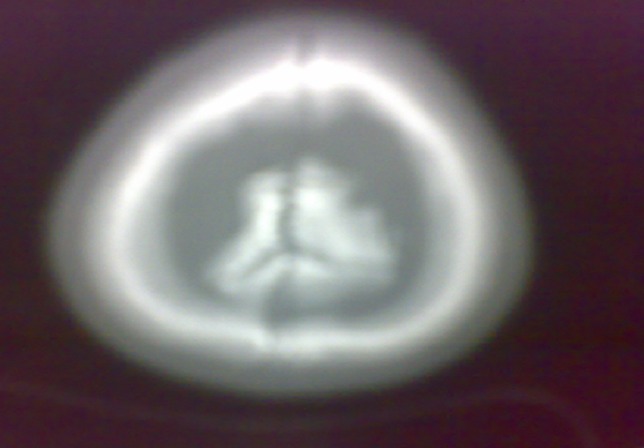
Bone window of coronal CT scan showing depressed fractures of bilateral parietal bones in high parietal region

**Figure 3:**
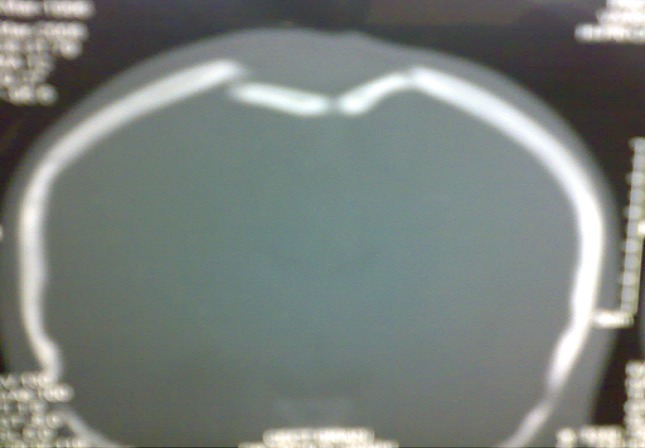
CT scan of the head showing bilateral depressed factures in high parietal area

On examination he had weakness of both lower limbs, with right sided affected more than left. Motor power on right lower limb was 3/5 and on left lower limb it was 4/5. Deep tendon reflexes were exaggerated and plantar reflex was extensor, on both the limbs. Computerized Topography (CT) scan of head was done which showed small bilateral depressed fractures of the high parietal bones (Figure 1). Symmetrically depressed fragments of parietal bones associated with hypodensities in the adjacent brain parenchyma, suggestive of edema were seen. However there was no evidence of associated injury around superior sagittal sinus and hematoma or contusion in the brain parenchyma (Figure 2, Figure 3).

The patient was managed conservatively for underlying associated brain oedema, around high cortex region and he responded well. His weakness progressively improved and he was discharged after 2 weeks without any sequelae.

## Discussion

Depressed skull fractures can occur in a variety of settings, depending upon several variables like the age, thickness of the vault, make of object, the force of the impact etc. The size of impacting device and the force of impact are directly related to the magnitude of the dynamic load. In this case, a wooden rod with large amount of kinetic energy made contact with small area over the skull leading to depressed skull fracture. Further, as in our case, initially there were no associated neurological manifestations of injury, so depressed fractures may be missed if radiological investigations are not done. Even careful clinical examination sometime does not reveal anything although complications and sequelae of depressed skull fractures can be minimised by early diagnosis [6]. The scalp is relatively mobile and any area of depression may not lie directly beneath the laceration. Visual inspection of the skull through the scalp laceration may fail to reveal a fracture. Careful digital exploration of the scalp wound with a gloved finger would reveal a bone edge, a depression, or a mobile bone fragment but this could not be performed in our case, as wound was just 1 cm in size. The management of choice in preventing infection from open depressed skull fractures is operative debridement and thorough irrigation, though there is evidence that selected cases can be safely managed without operation especially if the site of fracture is in proximity of the venous sinuses [7, 8]. Post trauma, brain edema after depressed fracture is usual phenomenon and patient can present as neurological deficit depending upon area of brain affected. However, local edema subsides usually after few days once active phase of injury is over [9].

## Conclusion

We conclude that, at times mild head injury can manifest as depressed skull fracture. Further, paraperesis is also a rare complication of depressed skull fracture.

## References

[1] Jennet B (1996). Epidemiology of head injury. J Neurol Neurosurg Psychiatry..

[2] Golfinos JG, Cooper PR, Cooper PR, Golfinos JG Skull fracture and post-traumatic cerebrospinal fluid fistula. Head Injury.

[3] Geisler FH, Wilkins RH, Regachary SS Skull fractures. Neurosurgery.

[4] Mehdi SA, Ahmed CB, Dogar IH, Shaukat A (2010). Depressed skull fracture; Interrelationship between CT evaluation of & its clinical findings. Professional Med J.

[5] Ishman SL, Friedland DR (2004). Temporal bone fractures: traditional classification and clinical relevance. Laryngoscope.

[6] Marion DW (1991). Complications of head injury and their therapy. Neurosurg Clin N Am.

[7] Van der Heever CM, Van der Merwee DJ (1989). Management of depressed skull fractures Selective conservative management of nonmissile injuries. J Neurosurg..

[8] Bullock MR, Chesnut R, Ghajar J, Gordon D, Harti R, Newell DW (2006). Surgical management of depressed skull fractures. Neurosurgery..

[9] Fuentes S, Metellus P, Levrier O, Adetchessi T, Dufour H, Grisoli F (2005). Depressed skull fracture overlying the superior sagittal sinus causing benign Intracranial hypertension Description of two cases and review of the literature. Br J Neurosurg.

